# Guided Imagery Relaxation in Quality of Life of Patients Undergoing Hematopoietic Stem Cell Transplantation: A Quasi-Experiment

**DOI:** 10.31557/APJCP.2021.22.8.2453

**Published:** 2021-08

**Authors:** Luana Aparecida Alves da Silva, Celina Angélica Mattos Machado, Edenice de Oliveira Santana, Mariana Nunes da Silva, Jorge Vinícius Cestari Felix, Namie Okino Sawada, Paulo Ricardo Bittencourt Guimarães, Luciana Puchalski Kalinke

**Affiliations:** 1 *Complex Hospital de Clinicas, Federal University of Parana, Brazil. *; 2 *Erasto Gaertner Hospital, Brazil. *; 3 *Federal University of Paraná, Brazil. *; 4 *Department of Nursing, Federal University of Parana, Brazil. *; 5 *Department of Nursing, University of São Paulo, Brazil. *; 6 *Department of Statistics, Federal University of Parana, Brazil. *

**Keywords:** Imagery, Psychotherapy, Relaxation Therapy, Hematopoietic Stem Cell Transplantation, Bone Marrow

## Abstract

**Objective:**

The aim of this study was to evaluate the effects of the relaxation technique with guided imagery by means of virtual reality on health-related quality of life in patients undergoing hematopoietic stem cell transplantation.

**Methods:**

A quasi-experiment conducted in a Bone Marrow Transplantation Service of a public hospital in southern Brazil. From October 2019 to October 2020, forty-two adult participants who underwent transplantation were included, 35 in the intervention group and seven in the control group. A guided imagery intervention, with audio guiding the relaxation associated with nature images in 360º, was performed during the hospitalization period. Data were collected on the first day of hospitalization, on the transplantation day, during the neutropenia stage, and at pre-hospital discharge. The Functional Assessment of Cancer Therapy-Bone Marrow Transplantation (FACT-BMT), Functional Assessment of Chronic Illness Therapy-Fatigue (FACIT-Fatigue) and Functional Assessment of Cancer Therapy-Neutropenia (FACT-N) were used to assess health-related quality of life, fatigue and neutropenia. Data were analyzed using the Generalized Linear Mixed Model for the evolution of the health-related quality of life assessments over time, considering the groups and stages. Pearson’s correlation coefficient was adopted for the correlation analyses.

**Results:**

Allogeneic transplantation was predominant: 28 (80%) in the intervention group and 5 (71.43%) in the control group. There were improvements in the health-related quality of life scores, although not significant. A significant difference was found among the stages (p<0.050) and a significant positive correlation (p<0.000) among the variables on general quality of life, additional concerns, fatigue and neutropenia in all stages.

**Conclusion:**

Patients undergoing hematopoietic stem cell transplantation suffer changes in their quality of life. Interventions based on integrative practices emerge as an option to minimize them.

## Introduction

Hematopoietic Stem Cell Transplantation (HSCT) is a complex and singular treatment, used for hematological and non-hematological diseases; the process requires the intervention of different health professionals. Studies in HSCT services show that the hospitalization period negatively affects the patient’s personal performance and causes additional concerns. These conditions generate an impairment in quality of life, which is conceptualized as multidimensional and includes physical, psychological and social domains (Brice et al., 2017; Machado et al., 2018).

Although the complications related to HSCT are difficult to control, it is the health professional’s duty, especially the nurse’s, to offer practical options aiming to reduce anxiety and the emotional, functional and social impacts, as well as to increase treatment effectiveness, thus improving health-related quality of life (HRQoL) (Liang et al., 2018). It is essential to assess HRQoL especially in Oncology, since the results indicate the consequences of the disease and treatment, its cost and benefits and the patient’s preferences. HRQoL can be defined as the perceived health status, that is, how much the disease or chronic state is perceived by the individual (Sawada et al., 2020).

Integrative and Complementary Health Practices (ICHPs) are options for interventions that promote HRQoL for patients with chronic diseases. They stimulate natural mechanisms for preventing harms and recovering health, and emphasize therapeutic bond and integration of the individual with the environment and society. They can help strengthen the immune system and relieve disease symptoms and adverse effects of conventional treatments (Liang et al., 2018; Carlson et al., 2017).

Among the ICHPs, mind-body therapies are focused on the interaction of brain, body, mind and behavior; as well as on the way in which emotional, mental, spiritual, experiential and behavioral factors affect health (NCCIH, 2018). They are used to create a feeling of relaxation and stress reduction, as well as to offer psychological and health benefits, including reduced symptoms of illness, improved coping, regulation of behavior, quality of life and well-being (Misra et al., 2019). 

Guided imagery relaxation therapy is based on the mind-body connection. It is a low-cost, safe and easy-to-apply intervention that leads the individual to create mental images which bring tranquility and comfort. The person receiving the therapy is invited to mentally elaborate a sequence of images to evoke one or more senses and access physical, emotional and spiritual dimensions influencing bodily change that help them feel calm, hope, happiness, contentment and relaxation, aiming to promote mental well-being (Carlson et al., 2017; Coelho, 2018).

The use of guided imagery relaxation can bring benefits in HSCT. The offer of complementary and integrative therapeutic options can contribute to the improvement of HRQoL, especially for those who are in hospital care and isolated from social activities. They can provide relief for physical and emotional symptoms, thus understanding the individual holistically.

Thereby, the aim of this research was to analyze the effects of a relaxation technique with guided imagery by means of virtual reality on health-related quality of life, in reducing fatigue and neutropenia in patients undergoing hematopoietic stem cell transplantation.

## Materials and Methods

This is a quasi-experimental, longitudinal and analytical study, with a non-equivalent control group (CG), conducted in a Bone Marrow Transplantation Service of a public teaching hospital in southern Brazil, from October 2019 to October 2020.

**Figure 1:**
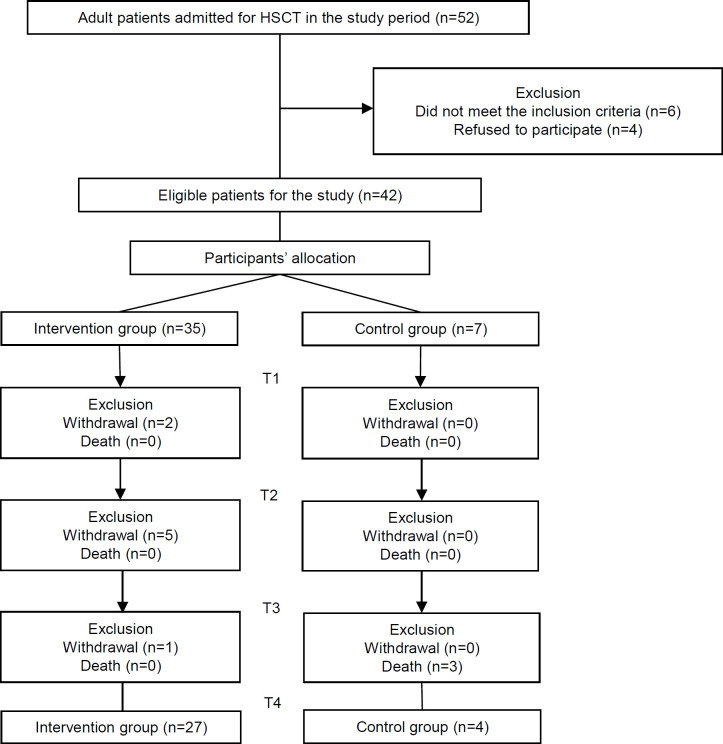
T1-Time 1 (First day of hospitalization); T2-Time 2 (Day zero, day on which transplantation was performed); T3-Time 3 (Neutropenia stage); T4-Time 4 (Pre-hospital discharge)

The sample size was calculated considering the annual mean of 52 HSCTs in adults, from 2016 to 2018. Complementing sample calculation, the mean score for Functional Assessment of Cancer Therapy-Bone Marrow Transplantation (FACT-BMT) of 108.4 with a standard deviation (SD) of 21.04, obtained in a study (Machado, 2017) conducted in the same service, was used. The total sample was composed of 42 participants, with an error margin of 5% and a confidence level of 95%; 35 were included in the intervention group (IG) and seven in the CG, allocated in a 5:1 ratio (Figure 1).

Patients aged 18 years old or older admitted for HSCTs were included. Those who were physically unable to complete questionnaires, see or hear were excluded, as well as those who had a history of or were being treated for vertigo, labyrinthitis and/or epilepsy with documented medical records. Patients who refused to continue receiving the intervention or to complete questionnaires, or those who stopped participating in two consecutive interventions, were discontinued.

The IG received the relaxation technique with guided imagery by means of virtual reality. The participant was offered virtual reality goggles with built-in headphones, then there was the reproduction of a background sound associated with a narration that guided the relaxation, followed by the projection of nature images in 360°. The technique lasted approximately 10 minutes and was applied three times a week from admission to hospital discharge.

Data collection occurred at four moments: at admission (T1); on day zero, on the transplantation day (T2); between days +5 and +8 during the neutropenia stage (T3), and at pre-hospital discharge (T4). A sociodemographic and clinical questionnaire (SDCQ), FACT-BMT version 4.0, Functional Assessment of Chronic Illness Therapy-Fatigue (FACIT-Fatigue) version 4.0, and Functional Assessment of Cancer Therapy-Neutropenia (FACT-N) version 4.0, were used.

In the Functional Assessment of Cancer Therapy-General (FACT-G), present in FACT-BMT and FACT-N, the domains on physical well-being (PWB), social/family well-being (SFWB) and functional well-being (FWB) show a variation in scores of 0-28, while the emotional well-being (EWB) domain varies from 0 to 24. The BMT subscale (BMTS) varies from 0 to 40, while the Neutropenia subscale (NS) from 0 to 76. The FACT-BMT scores vary from 0 to 148, the Trial Outcome Index - composed of the sum of the PWB, FWB and BMTS scores - varies from 0 to 96. In FACIT-Fatigue, the variation is 0-52. The higher the scores, the better the HRQoL.

All answers referring to SDCQ, FACT-BMT, FACIT-Fatigue and FACT-N were coded in Microsoft® Excel spreadsheets, Office 365®. The information was scanned twice independently and then validated. The Generalized Linear Mixed Model was adopted to analyze the evolution of the HRQoL scores over time for the groups and stages, considering an autoregressive covariance matrix of order one and defining the best fit through the Akaike Information Criterion. The correlation analysis for variables on General HRQoL (FACT-G), additional concerns, fatigue and neutropenia was performed by calculating Pearson’s correlation coefficient.

The research was approved by the Research Ethics Committee of the Clinical Hospital of the Federal University of Paraná, under number 3,446,872. It was registered in the Brazilian Registry of Clinical Trials, number RBR-37ymzb.

## Results

Of the initial sample of 42 participants, 31 (73.81%) completed the four moments of data collection, eight (22,86%) were discontinued in the IG, and three (42.86%) in the CG. In the first group, six participants became unable to continue due to treatment complications. In the CG, discontinuation was due to death. A mean of 11 interventions were performed per IG participant.

The general mean age was 37.8 years old, varying from 18 to 65. There was prevalence of allogeneic transplantation in 28 (80%) cases in the IG and 5 (71.43%) in the CG (Table 1).

In the HRQoL assessments, the PWB scores declined between T1 and T3, hospitalization and neutropenia, with a recovery at T4, pre-discharge, for both groups without returning to baseline values. For both groups, worse scores in FWB and EWB are observed at T1, indicating impairment even before the treatment was initiated. As the discharge period approaches after hematological recovery, there is an increase in the EWB scores when compared to the first assessment. For SWB and PWB, there is recovery, but the scores are still low. For BMTS and TOI, there is greater impairment during neutropenia, indicating worse HRQoL and an increase at T4, yet with lower scores when compared to T1, demonstrating the maintenance of HRQoL impairment during this stage (Table 2).

For FACT-G and FACT-BMT, there was a reduction in the scores at T2 and at T3 when compared to T1, and a subsequent recovery at T4, without reaching baseline values. The CG had worse scores at T3 and T4 in these assessments (Table 2).

There was no significant difference between the IG and the CG in PWB (p=0.721), SWB (p=0.470) and EWB (p=0.846), FACT-G (p=0.834) and FACT-BMT (p= 0.767), despite higher scores in the IG. However, a significant difference between treatment stages T1 and T3 (SWB - p=0.005; PWB, FACT-G and FACT-BMT - p=0.000) was found, except for EWB, for the total sample (Table 2).

For FACIT-Fatigue, there was no significant difference between the groups (p=0.496). Nevertheless, a significant difference was observed between stages T1 and T2 (p=0.032) and between T1 and T3 (p=0.000). It is possible to observe that the mean of the scores increases at the end of treatment at T4, with greater recovery in the IG when compared to the scores at T1, although there is no significant difference (Table 2).

Similarly to what was observed for fatigue, for NS there was no significant difference between the groups (p=0.469), only in the stages (p=0.000). Significant differences were observed between T1 and T2 (p=0.012) with worsening in the assessments, as well as from T1 to T3 (p=0.000) and from T2 to T3 (p=0.000), followed by an increase from T3 to T4 (p=0.001) with scores similar to T1 at pre-discharge. The scores increase at the end of treatment at T4, indicating better HRQoL. Moreover, greater recovery was observed in the IG when compared to T1 values, despite a non-significant difference between the groups (Table 2).

Regarding the correlation of variables for the IG, the results show a significant positive correlation, indicating an interaction among the FACT-G, FACT-BMT, FACIT-Fatigue and NS variables in all stages (Table 3). It can be inferred that impaired HRQoL interferes with impaired fatigue and neutropenia during all stages of HSCT. In other words, the decline and subsequent progressive recovery of HRQoL is related to fatigue and neutropenia during the HSCT process; the opposite is also true, which may influence HRQoL either positively or negatively.

**Table 1:** Sociodemographic and Clinical Characterization of the Participants Included in the Study

Variables	IG		CG		Total	
	n	%	n	%	n	%
Gender						
Male	22	62.86	4	57.14	26	61.90
Female	13	37.14	3	42.86	16	38.10
Marital status						
Married/Stable Union	17	48.57	3	42.86	20	47.62
Single	16	45.71	4	57.14	20	47.62
Widowed	1	2.86			1	2.38
Divorced	1	2.86			1	2.38
Family income						
No income	1	2.86			1	2.38
Up to US$ 210,00	3	8.57			3	7.14
Above US$ 210,00 and up to US$ 630,00	21	60	5	71.43	26	61.90
Above US$ 630,00 and up to US$ 2100,00	4	11.43	1	14.29	5	11.90
Above US$ 2100,00 and up to US$ 4200,00	1	2.86			1	2.38
Above US$ 4200,00	1	2.86			1	2.38
Did not know	4	11.43	1	14.29	5	11.90
Type of HSCT						
Allogeneic – related donor	15	42.86	2	28.57	17	40.48
Allogeneic – haploidentical donor	4	11.43	1	14.29	5	11.90
Allogeneic – unrelated donor	9	25.71	2	28.57	11	26.19
Autologous	7	20	2	28.57	9	21.43
Source of HST						
Peripheral blood	18	51.43	2	28.57	20	47.62
Bone marrow	16	45.71	5	71.43	21	50.00
Bone marrow and Peripheral blood	1	2.86			1	2.38
Diagnosis						
Leukemias	13	37.14	3	42.86	16	38.10
Lymphomas	7	20	1	14.29	8	19.05
Multiple myeloma	2	5.71	1	14.29	3	7.14
Myelodysplasia	1	2.86			1	2.38
Myelofibrosis	1	2.86			1	2.38
Severe Aplastic Anemia	9	25.71			9	21.43
Others	2	5.71	2	28.57	4	9.52

Note: US$ 210.00 is the approximate amount referring to the Brazilian minimum wage during the research period. IG, Intervention group; CG, Control group; HSCT, Hematopoietic stem cell transplantation; HST, hematopoietic stem cells.

**Table 2:** Comparison of Health-Related Quality of Life between Groups and Stages

Scales	T1		T1-T2	T2		T1-T3	T2-T3	T3		T3-T4	T4	
	IG(n=35)	CG(n=7)		IG(n=33)	CG(n=7)			IG(n=28)	CG(n=7)		IG(n=27)	CG(n=4)
	Mean±SD	Mean±SD	p	Mean±SD	Mean±SD	p	p	Mean±SD	Mean±SD	p	Mean±SD	Mean±SD
PWB	21.97±5.43	23.14±3.44	0.000	16.52±5.72	18±4.47	0.000	-	15.36±5.9	16.29±4.23	0.038	19.11±5.52	20±5.29
SWB	22.22±4.50	20.74±4.71	-	21.56±4.41	21.31±4.85	0.005	0.004	20.96±4.96	17.29±4.45	-	21.02±5.26	20,08±3.06
EWB	19,37±3.93	18.57±4.24	-	18.82±4.95	20±2.38	-	-	19.46±4	18.29±3.45	-	20±4.18	19.75±1.71
FWB	20.67±4.71	19.21±4.6	0.019	17.58±4.69	17.71±6.29	0.006	-	16.64±5.34	16.14±5.05	-	17.70±5.66	17.25±3.77
BMTS	29.03±4.95	28±4	-	24.85±5.4	26±3.37	0.001	-	24.04±4.53	23.14±4.06	0.011	26.22±5.21	25±6
TOI	71.67±11.97	70.36±9.39	0.000	58.94±13.9	61.71±12.26	0.000	-	56.04±13.3	55.57±12.45	0.030	63.04±14.56	62.25±14.41
FACT-G	84.24±13.76	81.67±11.51	0.015	74.47±14	77.03±13.56	0.000	-	72.43±14.62	60±14.71	0.010	77.84±16.22	77.08±11.68
FACT-BMT	113.27±17.69	109.67±15.21	0.023	99.32±18.48	103.03±15.99	0.000	0.047	96.46±18.36	91.14±17.53	0.006	104.06±20.72	102.08±16.85
FACIT-Fatigue	39.06±10.05	44.43±9.20	0.032	33.64±12.49	38.86±9.08	0.000	-	32.98±10.65	32.86±9.19	-	35.36±11.31	35±12.57
NS	48.39±11	58.20±11.44	0.012	45.69±12.73	49.64±11.04	0.000	0.000	39.23±12.66	39.27±11.31	0.001	45.44±12.55	47.97±11.78

Note: The p value refers to the comparison among stages for both groups (p<0.05). From T1-T4 and T2-T4, no statistical significance was found in any of the assessments. Legend: IG-Intervention group; CG-control group; SD-Standard deviation; PWB-Physical well-being; SWB-Social/family well-being; EWB-Emotional well-being; FWB-Functional well-being; BMTS-Bone Marrow Transplantation Subscale; TOI-Trial Outcome Index; FACT-G-Functional Assessment of Cancer Therapy-General; FACT-BMT-Functional Assessment of Cancer Therapy-Bone Marrow Transplantation; FACIT-Fatigue-Functional Assessment of Chronic Illness Therapy-Fatigue; NS-Neutropenia Subscale.

**Table 3:** Correlation between Overall Quality of Life, Additional Concerns, Fatigue and Neutropenia in the Intervention Group

		r	p	n	Intercept	Inclination
T1						
FACT-G	FACIT-Fatigue	0.628	0.000	35	50.67	0.86
	NS	0.551	0.000	35	50.86	0.69
FACT-BMT	FACIT-Fatigue	0.632	0.000	35	69.81	1.11
	NS	0.543	0.000	35	71.03	0.87
T2						
FACT-G	FACIT-Fatigue	0.76	0.000	33	45.96	0.85
	NS	0.77	0.000	33	35.78	0.85
FACT-BMT	FACIT-Fatigue	0.76	0.000	33	61.34	1.13
	NS	0.75	0.000	33	49.68	1.09
T3						
FACT-G	FACIT-Fatigue	0.65	0.000	28	43.18	0.89
	NS	0.6	0.000	28	45.47	0.69
FACT-BMT	FACIT-Fatigue	0.64	0.000	28	59.85	1.11
	NS	0.6	0.000	28	62.29	0.87
T4						
FACT-G	FACIT-Fatigue	0.75	0.000	27	39.81	1.08
	NS	0.71	0.000	27	36.07	0.92
FACT-BMT	FACIT-Fatigue	0.78	0.000	27	53.51	1.43
	NS	0.73	0.000	27	49.47	1.20

Legend: * p <0.000; r-Pearson’s correlation coefficient; p-probability of significance; SD-Standard deviation; FACT-G-Functional Assessment of Cancer Therapy-General; FACT-BMT-Functional Assessment of Cancer Therapy-Bone Marrow Transplantation; FACIT-Fatigue-Functional Assessment of Chronic Illness Therapy-Fatigue.

## Discussion

Relaxation therapy with guided imagery is a safe intervention option, with positive evidence, and which can be part of Nursing care plans promoting comprehensive care. 

The research participants are relatively young and of productive age, which is in line with the findings of other Brazilian studies that show means of approximately 38 years old (Machado et al., 2018; Azevedo et al., 2018). International studies show higher age means of the patients undergoing HSCT. Two South Korean studies have identified means over 48 years old (Kang et al., 2020; Kim et al., 2020). An Italian study found a mean of 50 years old (Biagioli et al., 2019), and a research study conducted in the United States found a mean of 56 years old (LaLonde et al., 2021). 

These patients are in the productive phase of their lives and, when faced with HSCT, they find themselves distanced from family, social and work life. Such factors generate negative impacts on all domains of HRQoL; the therapeutic course of cancer or HSCT has a strong economic impact with implications for the patient’s financial toxicity, when considering the loss of the ability to provide, in addition to the costs of treatment. 

In this study, the IG showed higher scores in the last two assessment stages, indicating better HRQoL. Nevertheless, the differences were not significant. A reduction in scores is observed on day zero and during pancytopenia, followed by a recovery at pre-discharge without reaching baseline values. Other studies assessing HRQoL using FACT-BMT at more than two time points during the HSCT period found similar results (Machado et al., 2018; Schumacher et al., 2018).

During hospitalization for HSCT, patients experience a series of symptoms that worsen with the progression of the treatment and a deterioration of their physical function. Pancytopenia is the most critical period, which is reflected in worse HRQoL, thus requiring more attention. After this period, hematopoiesis recovery and reduction of toxicity symptoms are related to an improvement in HRQoL scores (Liang et al., 2018). Nonetheless, changes in body image and in social and family relationships, as well as decreased functional capacity, do not allow the scores to return to pre-treatment values (Rivera-Fong et al., 2020).

The results found for EWB corroborate those found in another study, which assessed HRQoL in HSCT using FACT-BMT (Schumacher et al., 2018) and may be associated with the achievement of treatment goals and with hematological recovery, as well as with the prospect of hospital discharge, highlighting the importance of the patient’s emotional health during treatment. For SWB, greater impairment during pancytopenia was observed for the CG and less variation for the IG. In both groups, the scores at pre-discharge were close to the baseline. These results differ from Machado et al., (2018), who did not identify any recovery in the scores after pancytopenia, but corroborate the results of Schumacher et al., (2018).

No studies using guided imagery for HRQoL in HSCT were found for comparison. Using an exergaming intervention, a study found significant differences among the treatment stages and differences between the groups, with better results in the IG without significance (Schumacher et al., 2018). Another two studies that assessed HRQoL using the European Organization for Research and Treatment of Cancer Quality of Life Questionnaire Core 30 (EORTC QLQ-C30) and used interventions to improve HRQoL in HSCT did not find significant differences between the groups, a similar pattern to those found in the present research. The first used the Self Care Intervention in Oncology Nursing adapted for patients undergoing HSCT (Schmidt et al., 2017) and the second used music therapy (Tuinmann et al., 2017).

In Oncology, however, the use of mind-body techniques, such as relaxation with guided imagery, for patients in different treatment stages and survivors to improve HRQoL, has grown. Nicolussi et al., (2018) adopted guided imagery relaxation for cancer patients undergoing chemotherapy in southeastern Brazil assessing HRQoL using EORTC QLQ-C30. Significant differences were found for physical, emotional, cognitive and social function, role performance and fatigue, in addition to positive findings regarding symptoms in the assessment stages. 

A study conducted with Hispanic or Latino women diagnosed with cancer, using an intervention with guided imagery daily for 13 weeks, showed a significant improvement in EWB, but FWB and PWB were reduced; there was no significant difference between the groups (Hoogland et al., 2019).

In a study conducted with aged individuals with breast or prostate cancer that used guided imagery twice a day for six weeks, HRQoL was assessed with EORTC QLQ-C30, and it was shown that there was a significant increase in the IG immediately and 6 six weeks after the intervention in general quality of life and functional domains (Shahriari et al., 2017).

During pancytopenia, the patients experience the greatest cell depletion of all HSCT stages, combined with treatment toxicity, which is reflected in worse fatigue scores. In this research, similarly to HRQoL findings, the differences were not significant between the groups for fatigue. A significant difference was observed between the assessments at admission and pancytopenia. Nicolussi et al., (2018) found better IG fatigue scores. 

Differing from the results of the present research, two review studies point out that mind-body practices could reduce fatigue in cancer patients undergoing HSCT; relaxation was the best rated intervention for reducing fatigue (Duong et al., 2017; Hilfiker et al., 2018).

A randomized clinical trial using Benson’s relaxation response in HSCT showed that relaxation exercises significantly reduced the sensation of fatigue in the IG (Jafari et al., 2018).

Fatigue can also be related to erythrocyte and leukocyte changes in patients undergoing HSCT. NS underwent significant changes in the assessment stages, with the worst scores during pancytopenia, and with no difference between the groups. The period of severe leukopenia, neutropenia and thrombocytopenia that occurs shortly after HSCT infusion is expected, is the most critical in relation to the burden of symptoms for patients, and exerts a negative influence on HRQoL. In the present research, pancytopenia was the most critical period for the patients, evidenced by lower scores in all assessments. 

In this research, no significant differences were observed between the groups regarding HRQoL, fatigue and neutropenia using guided imagery relaxation as intervention. Cooley et al., (2013) suggest that the individual’s ability to generate and maintain mental images may interfere with the results; however, this attribute may have been mitigated with the use of virtual reality that does not depend on the participant’s imaginative capacity.

The dependence of HRQoL, fatigue and neutropenia is observed with a significant positive correlation among these variables. Corroborating these results, a study with patients undergoing chemotherapy identified a significant correlation between fatigue and HRQoL (Akin; Guner 2018). For these authors, the presence and severity of fatigue and neutropenia affect the capacity for self-care and decision-making, which influences a decline in HRQoL since it involves social, emotional and physical aspects.

For Cooley et al., (2013) a greater number of intervention sessions could lead to more favorable outcomes. In contrast, Hadjibalassi et al., (2018) state that the ‘dosage’ of integrative practices may not be measurable in terms of duration and that the conceptualization of effectiveness may not be uniform. In this research, it was not possible to assess if the frequency of the intervention could change the results.

Due to scarcity of studies involving guided imagery for HRQoL, in Oncology or HSCT, many of the practices adopted by researchers in the development of studies, such as script, duration, association with other techniques or number of intervention applications, show variations that make it difficult to generalize or compare the results.

The heterogeneity of the sample may have been a limiting factor. Other limitations include the number of discontinued participants, especially in the CG, despite being inevitable losses due to treatment complications, and the fact that the period of intervention application may not have been enough to influence HRQoL.

In conclusion, no evidence of improvement in HRQoL was observed in patients who received guided imagery relaxation. It was possible to observe that the pancytopenia stage is the most critical for patients undergoing HSCT, regarding losses in HRQoL, with significant differences in treatment stages for both groups assessed, where a positive correlation among HRQoL, fatigue and neutropenia was evidenced. 

The results point to the need to expand the options of ICHPs for patients undergoing HSCT in view of the impairment observed in HRQoL. In this sense, guided imagery relaxation is a safe, low cost and easy-to-apply option with the potential to help achieving the goals desired by hematological patients.

## Author Contribution Statement

Silva LAA, Santana EO and Kalinke LP conceived and designed the analysis. Guimarães PRB performed the statistical analysis. All authors discussed the results and contributed to the final manuscript. 
